# 

**Published:** 1886-10

**Authors:** 


### BOOKS RECEIVED.

*Causes de la Morbidité et de la Mortalité de la Première Enfance.* Par le Docteur Emil R. Coni, Imprimerie de Paul Emile Coni, Buenos Ayres.

*A System of Practical Medicine by American Authors.* Volume V. Diseases of the Nervous System. Philadelphia: Lea Brothers & Co. Chicago: A C. McClurg & Co.

*A Reference Hand-book of the Medical Sciences.* Volume III. New York: William Wood & Co. Chicago: W. T. Keener.

*Handbook of Practical Medicine.* By Dr. Hermann Eichorst. Volume II. Diseases of the Digestive, Urinary and Sexual apparatus. New York: William Wood & Co. Chicago: W. T. Keener.

*An Introduction to General Pathology.* By John Bland Sutton, F.R.C.S. Philadelphia: P. Blakiston, Son & Co. Chicago: W. T. Keener.

*The Genuine Works of Hippocrates.* Volume II. Translated by Francis Adams, LL.D. New York: William Wood & Co. Chicago: W. T. Keener.

*Diseases of the Stomach and Intestines.* By Professor Dujardin-Beaumetz. New York: William Wood & Co. Chicago: W. T. Keener.

*A Manual of Dietetics.* By J. Milner Fothergill, M.D., Edin. New York: William Wood & Co. Chicago: W. T. Keener.

*The Refraction and Accommodation of the Eye and their Anomalies.* By E. Landolt, M.D. Philadelphia: J. B. Lippincott Co. Chicago: W. T. Keener.

*A Manual of Practical Therapeutics.* By Edward John Waring. Fourth Edition. Philadelphia: P. Blakiston, Son & Co. Chicago: W. T. Keener.

*Manual of Differential Medical Diagnosis.* By Condict W. Cutler, M.S., M.D. New York and London: G. P. Putnam's Sons. Chicago: W. T. Keener.

*A Treatise on Electrolysis.* By Robert Amory, A. M., M. D. New York: William Wood & Co. Chicago: W. T. Keener.

*Index Catalogue of the Library of the Surgeon-General's Office, U. S. A.* Vol. VII.

*A Treatise on the Practice of Medicine.* By Roberts Bartholow, M. A., M. D., LL. D. Sixth Edition. New York: D. Appleton & Co. Chicago: A. C. McClurg & Co.

*Paralysis, Cerebral, Bulbar and Spinal.* By H. Charlton Bastian, M A., M. D., F. R. S. New York: D. Appleton & Co. Chicago: A. C. McClurg & Co.

*A Text-Book of Physiology.* By Dr. L. Landois. Philadelphia: P. Blakiston, Son & Co. Chicago: W. T. Keener.

*Rheumatism; its Nature, its Pathology and its Successful Treatment.* By T. J. Maclagen, M. D. New York: William Wood & Co. Chicago: W. T. Keener.

*A Manual of Minor Surgery and Bandaging.* By Christopher Heath, F. R. C. S. Philadelphia: P. Blakiston, Son & Co. Chicago: W. T. Keener.

*The Healing of Arteries After Ligature in Man and Animals.* By J. Collins Warren, M. D. New York: William Wood & Co. Chicago: W. T. Keener.

*Guy's Hospital Reports.* Vol. XLIII. London: J. & A. Churchill.

---

### PAMPHLETS RECEIVED.

*A Contribution to the Pathology of Hemianopsia of Central Origin.* By E. C. Seguin, M. D.

*Clinical Lectures on Orthopædic Surgery.* By A. Sidney Roberts, M. D.

*Report of the Delegates from the Philadelphia County Medical Society.*

*The Value of the Knee Phenomenon in the Diagnosis of Diseases of the Nervous System.* By Phillip Zeemer, M. D.

*Permanent Drainage for Ascites.* By Llewellyn Elliot, M. D.

*Medical Education and Medical Licensure.* By William H. Watson, A. M., M. D.

*Report of the Quarantine System of the St. Lawrence.*

*Meconeuropathia.* By C. H. Hughes, M. D.

*Quelques Cas D'Epilepsie.* Par le Dr. P. Glatz.

*Preliminary Analysis of the Bark of the Fonquieria Splendens.* By Helen C. De S. Abbott.

*Yucca Augustiflia.* By Helen C. De S. Abbott.

*Permanent Removal of Hair by Electrolysis.* By Samuel E. Woody, A. M., M. D.

*Intubation of the Larynx for Diphtheritic Croup.* By E. Fletcher Ingals, A. M., M. D.

*Impetigo Contagiosa.* By E. J. Beall, M. D.

*Electrolysis in Gynecology.* By Franklin H. Martin, M. D.

*The Non-Identity of Croupous Tonsillitis and Diphtheria.* By L. Emmett Holt, A. M., M. D.

*Simultaneous Double Amputation.* By Brice M. Hughes, M. D.

*Ligation of Internal Iliac.* By W. Locke Chew, M. D.

*Transactions of the Louisiana State Medical Society.* Eighth Annual Session.

SUBSCRIPTION PRICE, $3.00 PER YEAR, IN ADVANCE.

Whole Number, 321. OCTOBER, 1886. Vol. LIII. Number 4.

INDEX MEDICUS

LIBRARY INDEXED S.G.O.

THE

### CHICAGO MEDICAL JOURNAL AND EXAMINER

EDITORS:

S. J. JONES, M.D., LL.D.

W. W. JAGGARD, A.M., M.D. HAROLD N. MOYER, M.D.

COLLABORATORS.

CHICAGO:

PROFESSOR J. A. ALLEN, M. D., LL. D.
PROFESSOR E. ANDREWS, M. D., LL. D.
PROFESSOR W. H. BYFORD, A. M., M. D.
PROFESSOR O. C. DEWOLF, A. M., M. D.
PROFESSOR E. C. DUDLEY, A. B., M. D.
PROFESSOR J. H. ETHERIDGE, A. M., M. D.
PROFESSOR C. FENGER, M. D.
PROFESSOR J. N. HYDE, A. M., M. D.
PROFESSOR E. F. INGALS, A. M., M.D.
PROFESSOR H. A. JOHNSON, M. D., LL. D.
CHARLES GILMAN SMITH, A. M., M.D.

MILWAUKEE, WISCONSIN:
PROFESSOR N. SENN, M. D.

WASHINGTON, D. C.:
S. O. RICHEY, M. D.

UNITED STATES ARMY:
SURGEON, D. L. HUNTINGTON, M. D.

UNITED STATES NAVY:
MEDICAL DIRECTOR, J. M. BROWNE, M. D.
MEDICAL DIRECTOR, A. L. GIHON, A. M., M. D.

